# Magnetic Separation in Bioprocessing Beyond the Analytical Scale: From Biotechnology to the Food Industry

**DOI:** 10.3389/fbioe.2019.00233

**Published:** 2019-09-27

**Authors:** Sebastian P. Schwaminger, Paula Fraga-García, Marco Eigenfeld, Thomas M. Becker, Sonja Berensmeier

**Affiliations:** ^1^Bioseparation Engineering Group, Department of Mechanical Engineering, Technical University of Munich, Garching, Germany; ^2^Research Group Beverage and Cereal Biotechnology, Institute of Brewing and Beverage Technology, Technical University of Munich, Freising, Germany

**Keywords:** magnetic fishing, technical scale, industrial bioseparation, process design, protein purification, food technology, selective recovery

## Abstract

Downstream processing needs more innovative ideas to advance and overcome current bioprocessing challenges. Chromatography is by far the most prevalent technique used by a conservative industrial sector. Chromatography has many advantages but also often represents the most expensive step in a pharmaceutical production process. Therefore, alternative methods as well as further processing strategies are urgently needed. One promising candidate for new developments on a large scale is magnetic separation, which enables the fast and direct capture of target molecules in fermentation broths. There has been a small revolution in this area in the last 10–20 years and a few papers dealing with the use of magnetic separation in bioprocessing examples beyond the analytical scale have been published. Since each target material is purified with a different magnetic separation approach, the comparison of processes is not trivial but would help to understand and improve magnetic separation and thus making it attractive for the technical scale. To address this issue, we report on the latest achievements in magnetic separation technology and offer an overview of the progress of the capture and separation of biomolecules derived from biotechnology and food technology. Magnetic separation has great potential for high-throughput downstream processing in applied life sciences. At the same time, two major challenges need to be overcome: (1) the development of a platform for suitable and flexible separation devices and (2) additional investigations of advantageous processing conditions, especially during recovery. Concentration and purification factors need to be improved to pave the way for the broader use of magnetic applications. The innovative combination of magnetic gradients and multipurpose separations will set new magnetic-based trends for large scale downstream processing.

## Introduction

Many scientific discoveries are directly related to magnetic phenomena. From exploratory voyages using compasses to the development of electricity, and the processing of iron ores, magnetism has revolutionized traditional processes. Magnetic separation results from forces induced in magnetically susceptible materials by magnetic fields, while other materials are unaffected by such force fields. The first use of magnetic separation deriving from the mining industry goes back to the beginning of the twentieth century (Küster, 1902). With time, the areas of application were expanded to include coal desulfurization, steel production, wastewater treatment, medical applications, and biotechnology (Robinson et al., 1973; Whitesides et al., 1983; Moffat et al., 1994; Zhou et al., 1996; Yavuz et al., 2006; Gómez-Pastora et al., 2014; Egesa et al., 2017). What do all these applications have in common? The framework conditions, processing in a water medium, are similar. However, there are significant differences in the total volume processed as well as in process viscosities, which can cause challenges for magnetic separation. Biotechnological processes, where proteins or pharmaceuticals are the main products, deal with lower aqueous volumes, higher viscosity, and higher concentrations of target molecules compared to wastewater streams. Food technology processes are similar to those in biotechnology. In most cases, the targets are not magnetic. Based on different interactions between these targets and magnetic materials, they can be separated from their surrounding media. Medical applications such as *in vivo* hyperthermia or drug delivery treatments, deal with an even lower volume of water, higher purities, and lower toxicities. Thus, different magnetic materials are required depending on the underlying production processes and separation requirements. While the main goal of environmental approaches is to filter impurities and obtain clean water, in the life sciences, the aim is typically to remove only one target material from a mixture.

The materials used as carriers of biomolecules and the magnetic separator design have been further developed in the last years following new application trends. We review the industrially relevant magnetic separation processes for biotechnology and food technology with a focus on the advances of the last two decades. We show that the productivity levels achieved at larger scales are interesting for industrial exploitation. Perhaps the most pressing task at the moment is to encourage the development of enhanced devices for magnetic separation processes and to provide examples of optimal processing parameters. Novel processes are necessary to increase the productivity, recover more than one target material at a time and reduce time scales and water consumption. In the following sections, we want to highlight how magnetic separation can be used and is being used in the field of pharma and food industry and which parameters need to be considered in order to purify cells and biomacromolecules such as proteins.

## Advantages of Magnetic Separation

Conventional separation and purification approaches for pharmaceutical applications from biotechnological sources, such as the production of antibodies, require numerous steps: filtration, centrifugation, flocculation, sedimentation or crystallization, and chromatography techniques (Carta and Jungbauer, 2010). Developing high-tech or innovative approaches is still the principal challenge to promoting downstream processing in a leading technological field and to paving the way for enhanced productivity. Magnetic separation is an interesting candidate for future downstream applications due to some important advantageous features:

- Integrated one step capture and purification of target (high affinity and selectivity)
- High throughput
- Semi-continuous processing with low energy consumption.

Thus, magnetic separation can help reduce costs and increase yields and productivity compared to traditional processes. The continuous or semi-continuous processing at relatively low pressure leads to low processing energy costs. The process allows a broad framework of variables to adapt it to the necessities of each system and should lead to a higher number of bioproducts feasible for industrial exploitation (Hubbuch et al., 2001; Ohara et al., 2001; Ahoranta et al., 2002; Eskandarpour et al., 2009; Yavuz et al., 2009; Paulus et al., 2014; Gómez-Pastora et al., 2017).

## Separation Strategy and Separator Design

**Figure 1:**
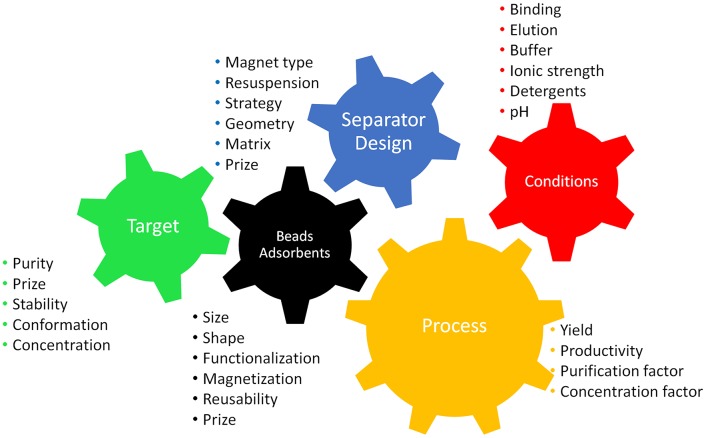
Scheme highlighting the main criteria for a magnetic separation process intermeshed like cogs in a machine as they are dependent on each other. Parameters need to be chosen according to the target product in order to facilitate an efficient process.

**Figure 2:**
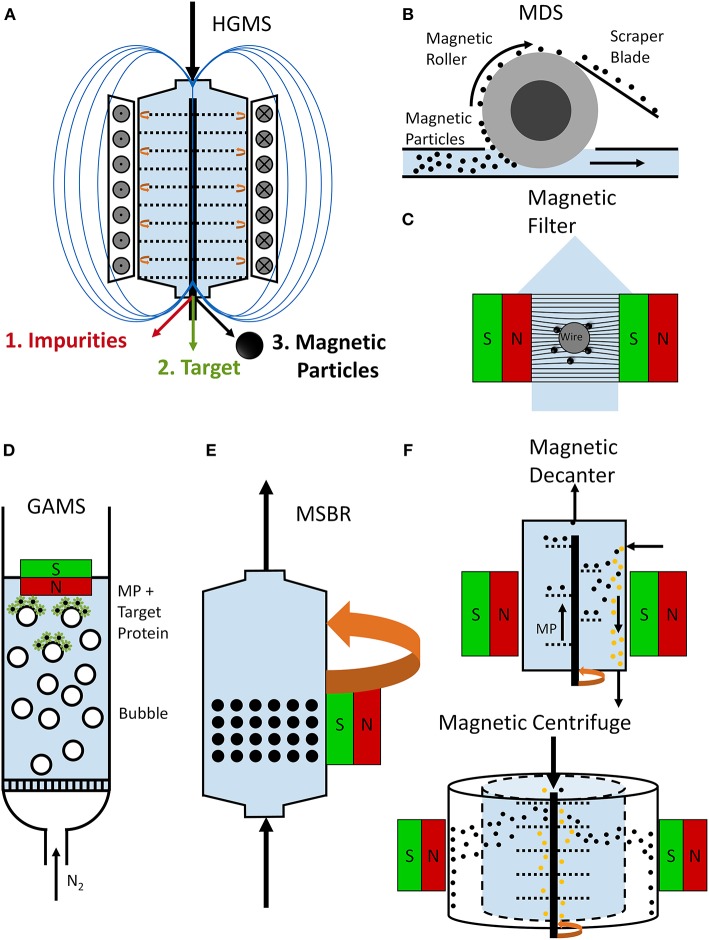
Schemes of magnetic separator designs. A rotor-stator high-gradient magnetic separator **(A)** can be used for the purification of target proteins. Here, an electro magnet is used to establish high magnetic field gradients between holey plates (rotor and stator plates). In a first step, the target material is adsorbed to the magnetic particles and separated magnetically from the impurities in the separation chamber. In a second step, the magnetic particles are separated from the target protein, which is eluted (Fraga García et al., 2015; Schwaminger et al., 2019a). An open-gradient magnetic separator (OGMS) **(B)** in a form of a magnetic drum separator (MDS) is illustrated. Magnetic beads are separated from impurities with a magnetic drum and recovered with a scraper blade (Dong et al., 2015). In a magnetic filtration set-up **(C)**, magnetizable wires, meshes, or bundles are placed in a magnetic field. Magnetic particles bind to these magnetizable matrices leading to a magnetizable filter cake, which improves the magnetic filter performance (Schwaminger et al., 2019b). During a gas-assisted magnetic separation (GAMS) process **(D)**, a gas is bubbled through the reactor leading to the flotation of magnetic particles and attached target molecules, which can be collected with a magnet (Li et al., 2013). A magnetically-stabilized moving bed reactor (MSBR) is based on a rotating magnetic field around the reactor, which allows a fluidization of magnetic beads while they behave like a fixed bed in flow direction **(E)** (Zong et al., 2013). A magnetic decanter **(F)** allows the continuous transport of magnetic particles with the magnetized screw while impurities are not affected by the magnetic field and thus separated from the magnetic material (Lindner and Nirschl, 2014). A magnetic centrifuge **(G)** allows a fast separation due to the density difference and the magnetization of magnetic particles (Lindner and Nirschl, 2014).

Before starting with a separation process, the first step is to select the most suitable separation strategy. This means thinking about the system and the process and taking into account all relevant parameters. The scheme in Figure 1 highlights the main criteria for designing an efficient process. It is necessary to bear in mind the processing constraints (volumes, targets, broth characteristics, time, costs, etc.) and the availability of suitable devices. Table 1 offers an overview of magnetic separation principles, while Figure 2 presents the set-up of some existing designs. The separation strategy is dependent on the target molecule and includes not only the actual magnetic separation process but also the interaction between the magnetic material and the target molecules. Conditions for binding and elution of target are crucial for the whole process and the equilibration times for binding and especially for elution still depict challenges for future optimizations.

**Table 1:** Summary of magnetic separation strategies for biotechnological downstream processing.

**Separation principle**	**Advantages**	**Disadvantages**	**Applications**	**References**
Magnetic collection	Fast, high throughput, process control	Recovery of MPs, multiple steps	Target purification (HGMS, OGMS)	Safarik et al., 2001; Brown et al., 2013; Fraga García et al., 2015; Müller et al., 2015; Schwaminger et al., 2019a
Magnetic flocculation	Fast, filtration	Inclusion of impurities, recovery, polymerbeads	Harvest	Svoboda, 1982; Wang et al., 2014
Magnetic flotation	Fast, separation, recovery	Aeration limitation, foaming, blockage	Purification, harvest (GAMS, GASE)	Li et al., 2013, 2014; Dong et al., 2015; Liu et al., 2016
Magnetic sedimentation	Separation, low loss of MP, characterization	Small scale, low density beads, energy (Centrifuge)	Magnetic centrifuge	Scherer et al., 2002; Berret et al., 2007; Lindner and Nirschl, 2014; Mykhaylyk et al., 2015
Magnetic sorting	Different shapes, sizes or magnetizations	Slow, small scale, expensive	Cell sorting	Miltenyi and Schmitz, 2000; Chen et al., 2017; Zhang et al., 2017; Solsona et al., 2018
Magnetic stabilized bed	Continuous, homogeneous bed	Pressure drop, reactor size, field circulation, diffusion	Processing (MSBR)	Albert and Tien, 1985; Rosensweig and Ciprios, 1991; Zong et al., 2013

*The advantages and the disadvantages of magnetic separation strategies are shown. The separator setups where these strategies can be applied are displayed*.

Several physical properties of magnetically susceptible materials are employed to separate molecules in a magnetic field (Moffat et al., 1994). The typical application of magnetic separation is the direct collection of magnetic materials and separating them from the non-magnetic ones. This approach uses high magnetic field gradients in order to successfully collect all magnetic material. Another option is to use the aggregation and agglomeration effects of magnetic materials due to the formation of magnetic dipoles in the presence of magnetic fields (Svoboda, 1982; Ditsch et al., 2005). This so-called magnetic flocculation can provide easier separation due to larger magnetic forces compared to the effect of the Stoke's drag force and Brownian motion; furthermore, this larger force also results in easier filtration due to the larger size of aggregates compared to single particles (Schwaminger et al., 2019b). The drawback is that this magnetic aggregation often negatively influences magnetic collection, making reuse of magnetic materials more challenging.

An interesting process, presented by Eichholz et al., is the magnetic filtration which combines magnetic separation with cake filtration (Eichholz et al., 2011). Other possibilities include magnetic flotation, enhanced magnetic sedimentation, magnetic sorting or the use of magnetic beads as adsorbent material in magnetically stabilized beds (Albert and Tien, 1985; Charles, 1990; Rosensweig and Ciprios, 1991; Moffat et al., 1994; Becker et al., 2009). Magnetic flotation can be used to collect or to enhance a flotation effect and separate and capture target molecules from impurities efficiently. The gas-assisted magnetic separation (GAMS) and the gas-assisted superparamagnetic extraction (GASE) process use nitrogen bubbles to float magnetic nanoparticles which are bound to target molecules (Li et al., 2013; Liu et al., 2016). The magnetic particles can be collected either in an extraction phase or with a magnet which allows for a fast separation process. Magnetic sedimentation can further be enhanced with a magnetic centrifuge which allows a faster separation by increasing the acceleration forces on the magnetic particles (Lindner and Nirschl, 2014). Magnetic sorting can be used to classify magnetic materials and materials bound to these magnetic materials according their fluidic as well as magnetic properties. This facilitates the sorting of different shapes and sizes of magnetic nanoparticles as well as the sorting of cells (Chen et al., 2017; Zhang et al., 2017). Magnetically stabilized bed reactors facilitate a hybrid between fixed and moving bed, which allows chemical reactions at the surface of magnetic beads where catalysts and enzymes can be immobilized (Zong et al., 2013).

## Magnetic Material

**Figure 3:**
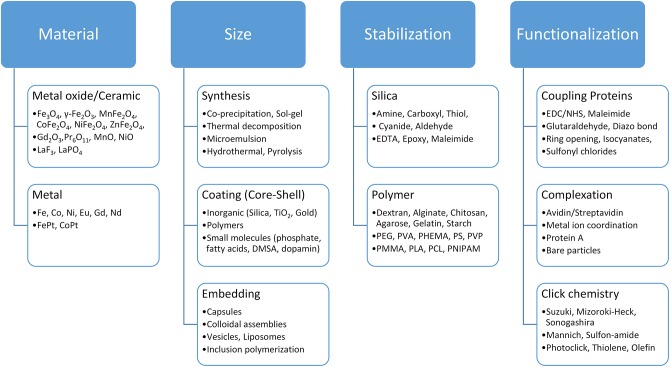
Toolkit for the selection of suitable magnetic beads according to the application. The choice of crude metal or ceramic particle and the strategy of stabilizing and functionalizing the magnetic particles play a decisive role for their application in bioseparation processes.

Magnetic material is an essential ingredient in the separation process. Magnetic separation has benefited especially from robust development in the medical technology sector (e.g., in drug delivery) as well as from the developments of multifunctional magnetic materials. Iron oxide nanoparticles are generally recognized as safe (GRAS) and have been approved by the US Food and Drug Administration (FDA) for *in vivo* applications (Thanh, 2012; Pušnik et al., 2016). Furthermore, iron oxides are allowed as color and food additives according to the FDA. There are multiple studies on the toxicity and possible health effects of iron oxide nanoparticles: On the one hand, iron oxide nanoparticles are able to penetrate cells and produce reactive oxygen species (ROS) which can cause cell damage (Soenen et al., 2011; Liu et al., 2013). On the other hand, multiple studies do not indicate health effects of iron oxide nanoparticles (Szalay et al., 2012). This controversy and thus the potential toxicity of iron oxide nanomaterials need to be further investigated and evaluated in order to ensure safe handling of nanomaterials for applications such as magnetic separation (Auffan et al., 2009; Valdiglesias et al., 2015). The synthesis of different magnetic particles, especially at the nano level, as well as a broad number of magnetic particle functionalization strategies, have been presented by numerous reviews (Bergemann et al., 1999; Berensmeier, 2006; Lu et al., 2007; Laurent et al., 2008; Philippova et al., 2011; Buck and Schaak, 2013; Conde et al., 2014; Xiao et al., 2016; Ge et al., 2017). The synthesis method influences both the particle core and its surface properties (Laurent et al., 2008; Shavel and Liz-Marzán, 2009; Roth et al., 2015). For iron oxides alone, multiple strategies ranging from co-precipitation to hydrothermal synthesis and from milling to biological synthesis are known (Laurent et al., 2008; Ali et al., 2016). Superparamagnetic nanoparticles can be used for separation processes as-synthesized or embedded in polymer matrices, often leading to microcarriers (Philippova et al., 2011). These microcarriers (Table 2) represent the most commonly used and commercially available particles for magnetic separation applications (Berensmeier, 2006; Borlido et al., 2013; Fields et al., 2016). Nanoparticles have an advantage over microparticle beads and chromatography resins as there is no mass transfer limitation for protein diffusion in the separation process, which is important in terms of capacity, purity, level of contamination and processing time. Non-embedded magnetic nanoparticles, such as bare iron oxide nanoparticles, have a great potential for separation processes due to their low production costs, the abundance of precursors, and their high density. On the other hand, magnetic microbeads are easier to handle in separation processes as they are less prone to magnetic aggregation effects. Thus, the larger size facilitates a better ratio between magnetic force and Stokes's drag force leading to a better severability in magnetic fields compared to colloidally stabilized nanoparticles. The selection of the optimal magnetic material depends strongly on the process strategy. Target biomaterial and process conditions also play a crucial role in the choice of the beads (see Figure 1). Depending on the prize and the requirements of the target product, either low-cost crude bare iron oxide nanoparticles or highly specific adsorbents, such as protein A modified magnetic particles, need to be used for purification processes (Holschuh and Schwämmle, 2005; Gomes et al., 2018; Schwaminger et al., 2019a,b). Furthermore, the conditions for binding and elution of the target protein must be accurately chosen with regard to the beads chemistry and process design. The functionalization of magnetic surfaces is a toolkit which can be adapted to the properties of the target and the selected switch conditions between adsorption and desorption. In summary, depending on the application, the magnetic material, the particle size, the stabilization and functionalization need to be selected and combined (Figure 3).

**Table 2:** Selection of commercially available magnetic beads for biotechnological purification and medical applications.

**Product**	**Size (μm)**	**Surface groups**	**Materials**	**Application**	**Manufacturer**
Dynabeads	1-4.4	Carboxyl, streptavidin, antibodies, antigens, DNA/RNA	ION + PS shell	Purification, analysis	Invitrogen
SiMAG	0.5-1	OH, COOH, SO_3_H, PO_3_H2, NH_2_, DEAE, PEI, C1, C2, C8, C18, Protein A, streptavidin, heparin	ION + SiOx	Purification, analysis	Chemicell GmbH
SPHERO	1-120	Amino, carboxyl, diethylamino, dimethylamino, hydroxyethyl	ION + PS shell	Purification, analysis	Spherotech, inc.
Pure proteome	0.3-10	Carboxyl, streptavidin, protein, N-hydroxy-succinimide (NHS)	ION + Polymer	Purification, sorting	Emd millipore
Pierce beads	1-10	Streptavidin, protein, NHS, antibodies, glutathione	ION + polymer	Purification, analysis	Thermo scientific
Sera-mag	1	Carboxyl, streptavidin, neutravidin, oligo amine, protein	ION + PS shell	Purification	GE lifescience
Biomag	1.5	Carboxyl, streptavidin, amine, antigen, antibody	ION + SiOx	Purification, medical	Polysciences, inc.
GenoPrep		Hydroxyl	ION + SiOx	Purification	GenoVision
MagaZorb	1-10	Hydroxyl	ION + Cellulose	Purification	Cortex biochem
MagneSil	5-8.5	Hydroxyl	ION + SiOx	Purification	Promega
MagPrep	1	Hydroxyl	ION + SiOx	Purification	Merck
MagSi	1-5	Hydroxyl		Purification	MagneMedics
MGP		Hydroxyl	ION + Pore free glass shell	Purification	Roche Diagnostics
M-PVA	0.5-8	PVA	ION + PVA	Purification	Chemagen
Sicastar	1-6	Maleic acid, Protein A + G, Carboxyl, Streptavidin, IDA/NTA	ION + PS-maleic acid copolymer	Purification	Micromod
BcMag	1, 5	Hydroxyl	ION + SiOx	Purification	Bioclone
BioMag	1	Hydroxyl	ION + SiOx	Purification	Bangs Lab
μMACS	0.05	Hydroxyl	ION + Dextran	Purification	Miltenyi
MPG	5	Hydroxyl	ION + Boro-silicate glass	Purification	PureBiotech
Nucleo-Adembeads	0.1-0.5	Hydroxyl	ION + Polymer	Purification	Ademtech
Scigen M	3.5	Hydroxyl	ION + Cellulose	Purification	Vector Lab
Feridex Combidex	0.015-0.2	Hydroxyl	ION + Dextran	Medical	Guerbet
Resovist Supravist	0.02, 0.06	Hydroxyl	ION + Carboxydextran	Medical	Schering
Clariscan Abdoscan	0.02, 3.5	Hydroxyl, Sulphonated styrene	ION + PEGStarch + SO_3_-PS-DVB	Medical	GE-Healthcare
VSOP-C184	0.007	Carboxyl	ION + Citrate	Medical	Ferropharm

## High-Gradient Magnetic Separation for Biotechnological Protein and Cell Recovery

High-gradient magnetic separators present large magnetic flux densities in the tesla range which allow for local gradients of 10^4^-10^5^ T/m or higher (Svoboda and Fujita, 2003; Las Cuevas et al., 2008). They enable the capture of materials with weaker magnetic moments from the flowing stream (Yavuz et al., 2009). Thus, emerging magnetic technology led to the development of different separation processes for biomolecules, especially proteins, at the laboratory scale. Nevertheless, only the work of a small number of research groups has been devoted to the larger scale development of magnetic separation for the recovery of proteins, other biomolecules and even cells (Hubbuch et al., 2001; Hoffmann et al., 2002; Hubbuch and Thomas, 2002; Bucak et al., 2003; Heebøll-Nielsen et al., 2004b; Hoffmann and Franzreb, 2004a,b; Moeser et al., 2004; Kampeis et al., 2009, 2011, 2013; Lindner and Nirschl, 2014; Nirschl and Keller, 2014; Fraga García et al., 2015; Roth et al., 2016; Gomes et al., 2018; Schwaminger et al., 2019a,b).

In the life sciences, high-gradient magnetic separation (HGMS) is “the integrated process consisting of coupling a batch-binding step to magnetic adsorbent handling (i.e., capture, washing, and elution) with a high-gradient magnetic filter” (Schultz et al., 2007). Hubbuch and colleagues observe that high-gradient magnetic fishing (HGMF) is the more complete name for the process (Hubbuch et al., 2001); however, HGMS is more commonly used in the literature. The last 20 years have seen a small revolution in HGMS for biotechnological applications. Overviews of process designs, device types and development as well as methods to determine processing relevant parameters have been presented (Franzreb et al., 2006; Schultz et al., 2007). We have gathered recently published articles focusing on the main challenges of magnetic separation in biotechnology and food technology and focus here on the studies that go beyond the laboratory scale (see Table 3). We want to emphasize the importance of binding and elution conditions in dependence of target molecules, purity, purification factor, yield, surface modification, and separator design. The equilibration times for binding and elution of the target molecule extensively contribute to the whole processing time with up to 2 h (Brechmann et al., 2019; Schwaminger et al., 2019a,b). Not only the time but the use of hazardous elution buffers such as imidazole and high concentration of elution buffers depict an ecological and economical challenge to magnetic separation processes (Schwaminger et al., 2019a).

**Table 3:** Summary of high gradient magnetic downstream processes at larger scales published since 2000.

**HGMS**	**Magnetic carrier**	**Broth characteristics**	**Target biomaterial**	**Feedstock volume/processing**	**P**	**Y**	**CF**	**PF**	**Further comments**	**References**
NdFeB magnet, 0.2 T, 2 L	Chitosan beads (47 μm, 65 μm)		Bovine trypsin	10 L						Safarik et al., 2001
Electro, 0.4 T	Bacitracin-linked beads (0.5-1 μm)	Cell-free *Bacillus clausii* broth	Savinase						Enzyme activity	Hubbuch et al., 2001
Electro, 0.4 T, 15 mL	Benzamidine-linked beads	Porcine pancreatin crude	Trypsin	0.4 L, 1 g/L beads in, 2 g/L beads out	62%		3.5			Hubbuch and Thomas, 2002
Electro, 0.4 T, 5 mL	Dextran beads	Filtered extract of jack beans	Concanavalin	125 mL, 4 g/L carrier	99%	69%		3.8		Heebøll-Nielsen et al., 2004a
Electro, 0.4 T, 5 mL	Cation-exchange beads	Clarified rennet bovine whey	Lactoperoxidase, lysozyme	380 mL, 2.5 g/L carrier		92%	4.7	36		Heebøll-Nielsen et al., 2004c
Electro, 0.4 T, 5 mL	Cation-exchange beads	Crude bovine whey	Lactoferrin, lacto-peroxidase, IgG	6 g/L					Fractionation (3 proteins)	Heebøll-Nielsen et al., 2004b
NdFeB magnet, 0.56 T, 4 mL	Cu-IDA beads	Crude sweet whey	Superoxide dismutase	52 mL,7 g/L beads in, 21 g/L beads out 0.15–0.6 g/L protein	86%		21			Meyer et al., 2005
NdFeB magnet, 0.32 T, 46 mL	Epoxy-PVA beads (1–2 μm)	*Candida antarctica*	Lipase						Multicyclic activity	Schultz et al., 2007
NdFeB magnet, 0.32 T, 182 mL	Functionalized beads	Crude sweet whey	Lactoferrin	2.2 L, 5 g/L carrier in		47%	1.7	18.6	Multicyclic recovery	Meyer et al., 2007
NdFeB magnet	DEAP beads	Clarified mare blood serum	Equine chorionic gonadotropin	0.5 L			5.4	975		Müller et al., 2011
Electro, R-S, 0.28 T, 160 mL	PAA beads (1.9 μm)	Filtered cheese, bovine whey	Lactoferrin, lactoperoxidase	10 L (multiple cycles), 2 L per batch, 2,5 g/L carrier	81 % (LPO)	2.3 (LPO)	73.4 (LPO)		Multicyclic recovery	Brown et al., 2013
Electro, R-S, 0.25 T, 980 mL	Cu-EDTA nano particles (22 nm)	Unclarified *E. coli* lysate	His-GFP	2.4 L, 100 g carrier, 22.3 g/L carrier in, 35 g/L carrier out, 8.5 g/L His-GFP	96%	93%	0.3	2.5	12 g/h; 2.2 g/L h	Fraga García et al., 2015
Electro, R-S, 0.25 T, 980 mL	DEAP beads	Pre-purified mare blood serum	Equine chorionic gonadotropin	20 L, 60 g carrier, 4.5 g/L carrier	56%	6.7	2049		Multicyclic recovery	Müller et al., 2015
NdFeB magnet, 0.56 T, 4 mL	Hydrophobic beads (0.8 μm)	Unclarified rabbit antiserum	Polyclonal antibody	11.6 mL, 2.5 g/L IgG, 9.3 g/L lysate, 31.7 g/L carrier out	81%	72%		3		Gomes et al., 2018
NdFeB magnet, 0.4 T, 122 mL	Bare Fe_3_O_4_ nano particles (12 nm)	Clarified *E. coli* lysate	Glu-GFP	1 L, 2 g, 2 g/L carrier 0.31 g/L lysate	68%	81%		2.1		Schwaminger et al., 2019b
Electro, R-S, 0.25 T, 980 mL	Bare Fe_3_O_4_ nano particles (12 nm)	Clarified *E. coli* lysate	His-GFP	2 L, 11 g, 5.5 g/L carrier, 1.5 g/L lysate	91%	38%		2.5		Schwaminger et al., 2019a
Electro, R-S, 0.25 T, 980 mL	Bare Fe_3_O_4_ nano particles (13 nm)	*S. ovalternus* cultivation	*S. ovalternus* microalgae cells	5 L, 0.3 g/L carrier, 0.6 g/L cells						Fraga-García et al., 2018
Electro, rod 1 T	Protein A agarose beads (90 μm)	CHO cell supernatant	Monoclonal antibody	26 L clarified cell-free harvest, 1.9 L bead for 5 g/L mAb		86%				Brechmann et al., 2019

*Separator data, carrier type and diameter, conditions, target material, process scale, and performance indicators are shown. Generally, the magnetic flux densities correspond to the values in the air gap and the volumes to the void values. Broth and target molecules data as well as the carrier data correspond to the ones in the process feed. In each case, the highest processing values were selected. Purity (P), yield (Y), purification factor (PF), and concentration factor (CF) represent generally the total process results and combine several elution fractions*.

Some fully automated HGMS models (Hubbuch et al., 2001; Hoffmann et al., 2002; Heebøll-Nielsen et al., 2004a; Hoffmann and Franzreb, 2004a,b; Meyer et al., 2005) and a series of patents from Franzreb and colleagues initiated a new era in high-gradient magnetic separation for large scale life science applications. In 2006 the so-called rotor stator separator was invented (Franzreb et al., 2004; Franzreb and Reichert, 2006). This model has the significant advantage of leading to substantially more efficient elution, washing and particle recovery steps. Thus, rotor-stator type separators can be used to upscale magnetic separation processes beyond conventional magnetic bead applications. Based on the rotor stator design (Franzreb and Reichert, 2006), some separator prototypes were built in collaboration with Steinert GmbH and Abbis GmbH. The prototype as well as the HGMS processing steps have been precisely described (Brown et al., 2013). A HGMS model has recently been adapted in collaboration with the Andritz KMPT GmbH to be cGMP compliant (Ebeler et al., 2018).

One interesting feature of the rotor stator separator is the simple design of multicyclic processes, which leads to higher overall yields, making it the better choice for high-value target biomolecules. Schultz et al. and Meyer et al. had previously presented multicycle protein recovery with other separators, but the challenges of resuspending particles, problems due to backmixing and incomplete flushing still remained (Meyer et al., 2005; Schultz et al., 2007). Müller and collaborators run a process with a rotor stator HGMS over 60 cycles with a very low loss of binding capacity (<10%) in the first 12 cycles; in one batch process, the authors achieve an average purification factor as high as 4,900 (Müller et al., 2011).

Pharmaceutically relevant proteins have been captured with magnetic separators by Holschuh and Schwämmle (2005). They purified antibodies with protein A-modified magnetic beads on a 100 L scale of cell culture supernatant. Recently, similar processing approaches have been improved and adapted for the rotor stator system (Müller et al., 2015; Gomes et al., 2018). These works make clear the relevance of HGMS for direct capture of biotargets at larger scales. Gomes et al. provide work on polyclonal antibody recovery from an unfiltered rabbit antiserum feedstock with a HGMS at a mini-pilot scale. They use 0.8 μm functionalized particles to recover antibodies from an initial antibody concentration of 2.5 g/L in the feed with a final total yield of ~72% in 0.5 h in a 3-fold purified form (Gomes et al., 2018). Müller purifies the glycoprotein equine chorionic gonadotropin (eCG) from up to 20 L horse serum and achieves concentration factors of ~7 in a multicyclic process with the rotor stator HGMS (Müller et al., 2015). In a recent study, Brechmann et al. demonstrate the purification of monoclonal antibodies from 26 L CHO cell supernatant with a HGMS and obtain the same purity as for a chromatography process (Brechmann et al., 2019).

Another important breakthrough on liter scale HGMS has recently been achieved using magnetic nanoparticles (instead of microparticles) for protein recovery (Fraga García et al., 2015). A mass as high as 100 g of coated nanoparticles has been applied successfully to recover 12 g His-GFP per hour from 2.4 L cell lysate. Until that moment, many researchers had argued that HGMS was not suitable for the separation of very small magnetic particles (<100 nm) (Kim et al., 2009), although the early work of Hatton's group had already demonstrated the suitability of coated nanoparticles in mL-scale processes (Bucak et al., 2003; Moeser et al., 2004). Two new works (Schwaminger et al., 2019a,b) provide evidence of the possibilities of HGMS using a material that is technically and industrially very interesting: low-cost bare iron oxide nanoparticles. Both works demonstrate the advantages of nanoparticle based liter-scale separation for achieving higher capacities. Furthermore, Schwaminger et al. also reveal that elution can be carried out without the need of hazardous and expensive eluents as imidazole. This is an evidence for the broad margin to enhancing results and moving toward more sustainable processing forms, which is expected to be one of the future focuses of downstream processing (Schwaminger et al., 2019a).

Another example of successful separation using bare iron oxide nanoparticles in liter scale HGMS to recover whole cells rather than proteins has also been published very recently (Fraga-García et al., 2018), although the basis for magnetic cell separation dates back to 1975 (Melville et al., 1975). The advantages of faster processing and high recovery yields in the case of cell separation were recognized several decades ago (Kronick et al., 1978), although the magnetic material was often used only to label the cells (Molday et al., 1977). More recent publications emphasize the larger scale possibilities for cell purification (Hultgren et al., 2004) and the relevance gained in the last two decades (Berger et al., 2001), which is being extended to the field of biomass harvesting (Hu and Hu, 2014; Fraga-García et al., 2018).

## Applications and Prospects for the Food and Beverage Industry

Magnetic nanoparticles can be used in the food industry as well. The removal of yeast in large fermentation processes for wine and beer processing are of great interest. Magnetic removal of yeast represents a cost-effective and simple process compared to traditional techniques, where yeast is frozen and exploded (Dauer and Dunlop, 1991; Berovic et al., 2014). Berovic et al. demonstrated a magnetic yeast removal process, which reduces the removal time from 60 days to 15 min while maintaining the taste standard (Berovic et al., 2014). Not only the removal but the entire fermentation process can be improved by immobilization of yeast cells on magnetic nanoparticles (Genisheva et al., 2011). Furthermore, the removal of haze, turbidity proteins, and unwanted flavors in wine is an interesting field of application (Safarik et al., 2007; Mierczynska-Vasilev et al., 2017). Polymer coatings on magnetic nanoparticles can be used specifically to remove proteins from wines without affecting taste and flavors (Mierczynska-Vasilev et al., 2017). Liang et al. have shown the removal of the unwanted byproduct 3-Isobutyl-2-methoxypyrazine using polymer-coated magnetic nanoparticles (Liang et al., 2018). Aside from the refinement of alcoholic beverages, immobilizing enzymes on magnetic nanoparticles can be used in the food industry for clarification of fruit juices (Mosafa et al., 2014).

Still another large area of interest for magnetic separation processes is the dairy industry. Several approaches for the separation and purification of whey proteins such as bovine serum albumin, lysozyme, lactoferrin, lactoperoxidase, α-lactoalbumin, and β-lactoglobulin exist at the laboratory scale and have been reviewed by Nicolás et al. (2019). While many studies look quite promising, we would like to highlight the processing scales beyond the milliliter scale. For the purification of whey, Heebøll-Nielsen et al. were among the first to introduce high-gradient magnetic fishing (Heebøll-Nielsen et al., 2004b). They were able to capture lysozyme as well as lactoperoxidase with magnetic cation-exchange beads from 375 to 174 mL of whey, respectively (Heebøll-Nielsen et al., 2004c). Further investigations of magnetic processing of whey were conducted by Meyer et al. (2005, 2007). They purified superoxide dismutase from ~50 mL of whey by HGMF with metal ion coordinated magnetic beads (Meyer et al., 2005). Furthermore, larger amounts of whey were introduced for the purification of the protein lactoferrin. Here, 2,200 mL of whey were processed with HGMF to purify 111 mg lactoferrin with polyglutaraldehyde coated silanized magnetic beads. A further processing step was the separation of lactoferrin from crude whey in a five cycle process with 2 L of whey feedstock batches (Brown et al., 2013). Similar magnetic separation approaches on the edge of food- and biotechnology are the purification of lectins from legume extracts and lysozyme from hen egg white (Heebøll-Nielsen et al., 2004a; Eichholz et al., 2011).

## Conclusion and Outlook

This review provides an insight into industrially relevant magnetic bioseparation processes. Moreover, we list all relevant factors to be taken into account for designing a magnetic separation process. We want to emphasize the still unexploited potential of magnetic separation techniques, which could be applied in industrial downstream processing for the pharmaceutical, nutritional, and medicinal sectors, among others. Magnetic separation might aid in overcoming the major challenges in downstream processing: (1) more sustainable bioprocessing and environmentally friendly elution media in the recovery steps; (2) reduced water volumes to enhance concentration factors and decrease water consumption. Magnetic separation can be implemented as a direct capture and concentration step from crude cell broths. Additional techniques might be necessary to further polish proteins and improve the purity of pharmaceutical target products. However, the target requirements, the magnetic adsorbents, the processing conditions, and the separator design affect each other.

Most investigations on the enhancement of chromatography materials as well as magnetic beads seek only to improve the binding behavior. Hence, a better understanding of the adsorption mechanisms but more importantly of the desorption steps is necessary. While there is a great acceptance for chromatography in industrial downstream processes, magnetic separation needs to be established as an alternative. Here, regulations from the FDA for iron oxides as food additives and their use in medical applications will ease the acceptance for industrial bioseparation processes as well. The most important factor may be to encourage the design and engineering of improved systems for different biotechnological goals. At the moment, choosing a device for magnetic based separation of biomolecules is difficult. More effort must be devoted to the development of modern apparatuses by learning from cutting-edge technologies to apply less conservative but more dynamic industrial approaches. This might be the greatest challenge for establishing magnetic separation as an industrial alternative to conventional purification methods in the first and middle stages of downstream processing applications.

Magnetic separation systems are robust and the running costs are low. The change from magnetic microbeads, which are commonly used in magnetic separation processes, to nanoparticles with an even higher specific surface area and lower production costs might pave the way for even more propitious processing strategies. Moreover, the setup designs, which can be quite simple, lead to processing with low complexity. These advantages should lead to innovative, industrially appealing processes for large scale downstream processing in the future. Magnetic separation can also entail greater productivity and lower product prices for target molecules, thereby extending the number of products of biotechnological origin. In conclusion we would like to encourage more research and technical processing using magnetic forces, particularly for other life science fields such as the food and beverage sector.

## Author Contributions

SS and PF-G planned and designed the manuscript. SS, PF-G, and ME collected and reviewed the literature. SB and TB discussed the manuscript. The manuscript was written through contributions of all authors. All authors have given approval to the final version of the manuscript.

### Conflict of Interest

The authors declare that the research was conducted in the absence of any commercial or financial relationships that could be construed as a potential conflict of interest.
